# Association of selenium intake with bone mineral density and osteoporosis: the national health and nutrition examination survey

**DOI:** 10.3389/fendo.2023.1251838

**Published:** 2023-09-29

**Authors:** Shiyu Peng, Gaoxiang Zhang, Decheng Wang

**Affiliations:** Department of Spinal Trauma, Beijing Tongzhou District Hospital of Integrated Traditional Chinese and Western Medicine, Beijing, China

**Keywords:** selenium intake, bone mineral density (BMD), osteoporosis, NHANES, cross-sectional survey

## Abstract

**Background:**

Osteoporosis (OP) is a systemic metabolic skeletal disorder characterized by a decrease in bone mineral density (BMD) and an increase in the risk of fracture. The level of selenium (Se) in serum is associated with BMD. However, the relationship between dietary and total selenium intake and parameters such as osteoporosis and BMD is unclear. By conducting National Health and Nutritional Examination Surveys (NHANES), in this study, we assessed the association of Se intake with BMD and the risk of OP among general middle-aged and elderly people.

**Methods:**

The data were collected from three cycles of NHANES [2009–2010, 2013–2014, and 2017–2020]. Information on the dietary and supplementary Se intake was obtained from 24-h dietary recall interviews. Additionally, dual-energy X-ray absorptiometry (DXA) was performed to measure BMD, which was later transformed into T-scores; OP was diagnosed when the T-score was ≤ -2.5. We constructed a logistic regression model for the association between selenium intake and the risk of OP based on the estimated odds ratios (ORs) and the 95% confidence intervals (CIs). We also constructed a multivariable linear regression model to analyze the relationship between selenium intake and BMD.

**Results:**

In this study, 3,250 individuals (average age: 60.01 ± 10.09 years; 51.88% females) participated. The incidence of OP was 9.35% (3.30% for males and 17.75% for females). In the logistic regression model adjusted for every interested covariate, a higher quartile of dietary Se intake (OR for quartile 4 *vs*. quartile 1: 0.63; 95% CI: 0.41–0.96; P for trend = 0.027) was related to a lower risk of OP relative to the lowest quartile. The total selenium intake also exhibited a consistent trend (OR for quartile 4 *vs*. quartile 1: 0.67; 95% CI: 0.44–1.01; P for trend = 0.049). The results of the adjusted multivariate linear regression model showed that the participants with the highest quartile of dietary Se intake (Q4) had higher BMD in the total femur (β = 0.069, P = 0.001; P for trend = 0.001), femoral neck (β = 0.064, P = 0.001; P for trend = 0.001), and total spine (β = 0.030, P = 0.136; P for trend = 0.064) compared to those in quintile 1 (Q1). A similar trend of associations was observed for the total selenium intake with BMD, which was more prominent among females, as determined by the subgroup analysis.

**Conclusion:**

In this study, the dietary intake and total intake of selenium were positively associated with BMD, whereas they were negatively associated with the risk of OP among adults in the US. Further studies are required to verify our results and elucidate the associated biological mechanism.

## Introduction

Osteoporosis (OP) is a commonly occurring skeletal disorder and is characterized by a decrease in bone mass, low bone mineral density (BMD), and the deterioration of bone microstructure (1, 2). A study found that in 2010, OP affected 10.2 million adults in the US who were ≥ 50 years old. The number of affected individuals might reach 13.5 million in 2030 (3). OP significantly increases the burden on the social healthcare system because of the high morbidity, mortality, and therapeutic expenses associated with it (4, 5). Various risk factors are associated with the occurrence of OP and a decrease in BMD; these factors include genetic, environmental, and dietary factors (6, 7). Dietary factors are considered to be closely associated with musculoskeletal diseases (8). Some studies have shown that macronutrients (carbohydrates, proteins, and lipids), flavonoid polyphenols, and micronutrients (phosphorus, calcium, magnesium, and vitamins D, C, and K) greatly facilitate the inhibition of osteoporosis (9). Deficiency or excess of zinc, copper, selenium, iron, cadmium, silicon, and fluorine might affect bone mineralization and lead to osteoporosis (10). Some studies have shown that trace elements can help in preventing OP (11, 12).

Selenium (Se) is an essential trace mineral element in the human body. It forms “selenoproteins” after it is incorporated into the protein polypeptide chain. Additionally, Se modulates cell processes by influencing Se-mediated antioxidant enzyme components, which scavenge reactive oxygen species (ROS) in cells. Selenium is closely associated with Keshan disease, thyroid-related diseases, and other functions and diseases (13, 14). Selenium protein P is a critical Se transporter in the bone and is very important for maintaining bone health (15). A deficiency of Se may increase the levels of ROS, and it is the most important cause of OP (16). Some studies have suggested that serum Se level is positively associated with bone outcomes, like BMD and the risk of fracture (17, 18). Only a few studies have shown a non-linear relationship (19) and an inverted U-shaped trend (20) between the dietary intake of Se and osteoporosis. Although recent studies on humans have analyzed the relationship between the dietary Se level and BMD (20), few studies have investigated the relationship of dietary and total Se with BMD and osteoporosis. Hence, the effects of selenium intake on an increase in BMD and a decrease in the risk of osteoporosis need to be investigated to develop more effective preventive measures for reducing the morbidity and mortality of OP globally.

In this study, we investigated the relationship between Se intake and changes in BMD and the risk of OP based on the cross-sectional data collected by conducting National Health and Nutritional Examination Surveys (NHANES).

## Materials and methods

### Participants

**Figure 1:**
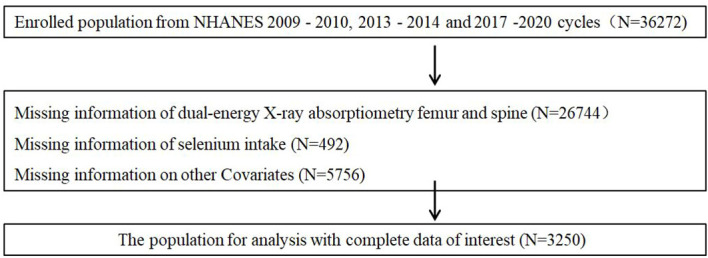
Flow diagram of inclusion criteria and exclusion criteria.

The NHANES was developed in 1959 as a continuous program for conducting cross-sectional examinations of the nutrition and health of people in the United States (US) annually. In this program, information on dietary, demographic, questionnaire, and laboratory data is released every two years. In this study, we included adults who were ≥50 years old, with sufficient selenium intake, and with BMD data recorded in the NHANES that was conducted in 2009–2010, 2013–2014, and 2017–2020 (**Figure 1**). All participants provided informed consent for this survey, as it was required by the Institutional Review Board of the National Center for Health Statistics.

### Selenium intake

The information on the dietary and supplementary selenium intake was collected from two 24-h dietary recall interviews in three NHANES two-year cycles (2009–2010, 2013–2014, and 2017–2020). It was a respondent-driven approach to collect precise and comprehensive data on the food and beverage types and amounts (every water type was included) taken within 24-h immediately before the interview (midnight to midnight). In this survey, two 24-h dietary recalls were conducted; the first one was conducted in person during the mobile examination, whereas the second one was conducted after 3–10 days through a telephone interview. Information on the dietary selenium intake and supplement intake was obtained in each interview; thus, the total selenium intake for each interview was the sum of dietary selenium and supplements. The average selenium intake recorded in two consecutive interviews was considered to be the selenium intake of the participants. A nutrient residual model was used to adjust the selenium intake for total energy intake (21). The approach helped adjust the total energy intake-induced confounding when analyzing the relationship between selenium and OP. Dietary Reference Intakes: Basic Guidelines for Nutritional Requirements (2006) recommended dietary reference intakes (DRIs) of total selenium intakes as follows: an estimated average intake (EAR) of 45µg/day, a recommended dietary allowance (RDA) of 55µg/day, and a tolerable maximum intake (UL) of 400µg/day, without gender differences.

### Determination of BMD and the diagnosis of OP

The BMD levels were examined at different regions (total femur, femur neck, and lumbar spine) in the NHANES. Additionally, through dual-energy X-ray absorptiometry (DXA), Hologic QDR-4500A fan-beam densitometers (Hologic, Inc., Bedford, Massachusetts) were used for scanning. Generally, the left hip was examined, only if the subject reported a fracture, a pin, or a replacement of the left hip, the right hip was examined. The BMD of the lumbar spine was determined by the average level of the first to fourth lumbar vertebrae. Pregnant patients, those with a radiographic contrast material history, those with bilateral hip fractures/replacement/pins, and those who were >450 lbs were eliminated from the DXA examination. The diagnostic criteria for osteoporosis were defined using the mean of the peak BMD of the femoral neck, total hip, and lumbar spine from the NHANES III database in the United States and its corresponding standard deviation (SD) in white women aged 20 to 29 years. Then, an established method was used to transform the BMD levels in the femoral neck, total hip, and lumbar spine to T-scores (22, 23), with a T-score≤ -2.5, -2.5 < T-score ≤ -1, and > –1 indicating OP, osteopenia, and normal, respectively. Patients with osteopenia and normal individuals were considered to be non-OP.

### Covariate assessment

Based on the statements on the NHANES website, trained personnel collected the data at every study site following standard procedures. In this study, the covariates were age, gender, ethnicity, education level, marital status, history of smoking, hypertension, diabetes, osteoporosis in parents, previous fractures, and body mass index (BMI, which was determined by dividing the body weight (kg) by the body height squared (m^2^). Participants were weighed in kilograms using a digital weight scale. Standing height was measured using a stadiometer with a fixed vertical backboard and an adjustable head piece.

### Statistical analysis

The estimates of the people in the US were generated by data weighting and analyzed by layering and clustering. For descriptive analysis, normally-distributed continuous data were represented by the mean ± standard deviation (SD), whereas non-normally distributed continuous data were expressed as the median and the quartile range. Additionally, categorical data were represented by the frequency and frequency percentage. We conducted the Student’s t-test, non-parametric tests, and the Chi-squared test to analyze the statistical differences between the two groups of continuous variables that followed a normal distribution, continuous variables that did not follow a non-normal distribution, and categorical data, respectively. The dietary and total selenium intake levels were classified according to quartiles (quartiles 1–4 indicated <25^th^, 25^th^–50^th^, 50^th^–75^th^, and >75^th^ percentile). Odds ratios (ORs) and 95% confidence intervals (CIs) were determined through logistic regression to analyze the relationship between selenium intake and the risk of OP. We used dietary and total selenium intake for reference. In Model 1, sex, body mass index, marital status, and race were adjusted. In Model 2, a history of hypertension, diabetes, osteoporosis in parents, and previous fractures were also adjusted. In Model 3, every interested covariate was adjusted. Additionally, multivariate linear regression models were constructed to determine the relationship between selenium intake and BMD in the total spine and total femur. The relationships between selenium intake and BMD, as well as the risk of OP were also analyzed through a gender-stratified subgroup analysis. The R (version 3.5.3) and SPSS (version: 24.0; SPSS, Chicago, IL) software programs were used for conducting statistical analyses. All differences among and between groups were considered to be statistically significant at P < 0.05 (two-sided).

## Results

### Characteristics of the study population

The unqualified patients were excluded (**Figure 1**), and 3,250 participants were included in this study. The characteristics of the participants with and without OP are presented in **Table 1**. Compared to the participants without OP, those with OP were thinner and older, mainly women, with lower levels of education, widowed, divorced, or separated, with hypertension, a history of osteoporosis in parents, previous fractures, and less likely to be non-Hispanic Black. In contrast, participants without OP had a greater amount of dietary Se and total Se.

**Table 1:** Basic characteristics of the OP and non-OP population of NHANES.

Variables	No osteoporosis	Osteoporosis	P
Number, n(%)	2946(90.65%)	304(9.35%)	
Age (years)	52.00(59.00,66.00)	66.00(59.00,75.00)	<0.001
<60 ,n(%)	1519(51.6)	78(25.7)	<0.001
≥60 ,n(%)	1427(48.4)	226(74.3)	
BMI (kg/m^2^), n(%)			
<25	699(23.7)	165(54.3)	<0.001
25-30	1143(38.8)	93(30.6)	
>30	1104(37.5)	46(15.1)	
Sex, n (%)			
Male	1514(51.4)	50(16.4)	<0.001
Female	1432(48.6)	254(83.6)	
Race, n (%)			
Mexican American	420(14.3)	40(13.2)	<0.001
Other Hispanic	315(10.7)	43(14.1)	
Non-Hispanic White	1243(42.2)	147(48.4)	
Non-Hispanic Black	675(22.9)	28(9.2)	
Other race	293(9.9)	46 (15.1)	
Education, n (%)			
Less than 9th grade	260(8.8)	44(14.5)	0.002
9–11th grade	330(11.2)	40(13.2)	
High school	673(22.8)	79(26.0)	
Some college	910(30.9)	75(24.7)	
College graduate	773(26.2)	66(21.7)	
Marital status, n (%)			
Married or living with partner	1922(65.2)	164(53.9)	0.012
Widowed, divorced or separated	791(26.8)	122(40.1)	
Never married	233(7.9)	18(5.9)	
History of smoking			
No	1627(55.2)	186(61.2)	0.052
Yes	1319(44.8)	118(38.8)	
History of hypertension, n (%)			
No	2232(75.8)	184(60.5)	<0.001
Yes	714(24.2)	120(39.5)	
History of diabetes, n (%)			
No	2461(83.5)	264(86.8)	0.136
Yes	485(16.5)	40(13.2)	
History of osteoporosis in parents, n (%)			
No	2556(86.8)	243(79.9)	0.001
Yes	390(13.2)	61(20.1)	
History of previous fractures, n (%)			
No	2661(90.3)	253(83.2)	<0.001
Yes	285(9.7)	51(16.8)	
Intake of selenium			
Total selenium (µg/day)	111.73(80.34,149. 41)	93.03(70.26,127.00)	<0.001
Dietary selenium (µg/day)	100.53(74.49,132.60)	86.30(62.84,107.18)	<0.001
Bone mineral density			
Total femur (gm/cm^2^)	0.96(0.14)	0.70(0.10)	<0.001
Femoral neck (gm/cm^2^)	0.80(0.13)	0.57(0.08)	<0.001
Total spine (gm/cm^2^)	1.04(0.15)	0.75(0.10)	<0.001

Categorical variables are presented as frequencies (%); continuous variables with normal distribution are shown as means (SDs); continuous variables with a skewed distribution are shown as medians (inter-quartile ranges).

### Relationship between selenium intake and osteoporosis

The relationship between selenium intake and the risk of OP was analyzed by conducting logistic regression (**Table 2**). In Model 1, based on the ORs and 95% CIs for the relationship between the dietary intake of Se with the risk of OP, after being adjusted for sex, body mass index, marital status, and race, a higher quartile of dietary selenium intake (0.69 (95% CI: 0.49, 0.98); 0.49 (95% CI: 0.33, 0.74); P for trend = 0.001) indicated a lower risk of OP. Additionally, dietary selenium intake showed consistent results with marginal significance (0.53 (95% CI: 0.35, 0.81); P for trend = 0.002; 0.63 (95% CI: 0.41, 0.96); P for trend = 0.027) after further adjustment for history of hypertension, diabetes, OP in parents, previous fractures (Model 2), and every interested covariate (Model 3). For the relationship between the risk of OP and the total selenium intake, consistent findings were obtained from Models 1, 2, and 3, i.e., 0.58 (95% CI: 0.39, 0.86) (P for trend = 0.005) after being adjusted for sex, BMI, marital status and race (Model 1); 0.60 (95% CI: 0.40, 0.90) (P for trend = 0.012) after further adjustment for history of hypertension, diabetes, OP in parents, and previous fractures (Model 2); 0.67 (95% CI: 0.44, 1.01) after being adjusted for every covariate (Model 3). Relative to the lowest quartile, the dietary and total Se intake did not show a significant inverse relationship in quartile 2.

**Table 2:** Odds Ratio of osteoporosis across quartiles of selenium intakes.

Intake of selenium (µg/day)	Range of selenium intake (µg/day)	Model 1^a^		Model 2^b^		Model 3^c^	
		OR (95% CI)	P-value	OR (95% CI)	P-value	OR (95% CI)	P-value
Dietary selenium (µg/day)							
Q1	≤73.15	Reference	–	Reference	–	Reference	–
Q2	73.16-98.55	0.83(0.60,1.14)	0.248	0.83(0.60,1.15)	0.269	0.88(0.63,1.22)	0.434
Q3	98.56-130.05	0.69(0.49,0.98)	0.036	0.72(0.51,1.03)	0.069	0.80(0.56,1.15)	0.230
Q4	≥130.06	0.49(0.33,0.74)	0.001	0.53(0.35,0.81)	0.003	0.63(0.41,0.96)	0.031
P for trend		0.001		0.002		0.027	
Total selenium (µg/day)							
Q1	≤79.35	Reference	–	Reference	–	Reference	–
Q2	79.36-110.05	0.91(0.66,1.23)	0.572	0.91(0.66,1.27)	0.582	0.98(0.70,1.37)	0.899
Q3	110.06-147.75	0.79(0.56,1.12)	0.184	0.83(0.58,1.17)	0.285	0.88(0.62,1.23)	0.500
Q4	≥147.76	0.58(0.39,0.86)	0.007	0.60(0.40,0.90)	0.013	0.67(0.44,1.01)	0.057
P for trend		0.005		0.012		0.049	

a Model 1 is shown as the odds ratio (95% confidence interval); adjusted for sex, body mass index, marital status and race.

b Model 2 is shown as an odds ratio (95% confidence interval); further adjusted for history of hypertension, history of diabetes, history of osteoporosis in parents and history of previous fractures.

c Model 3 is shown as odds ratio (95% confidence interval); further adjusted for education ,age and history of smoking .

### Association of selenium intake with BMD

The results of the multivariate linear regression analysis of selenium intake with BMD are presented in **Table 3**. The associations between selenium intake and BMD at various bone sites were analyzed (**Table 3**). In the crude model, relative to the lowest quartile (Q1) of dietary selenium intake, individuals with the highest quartile (Q4) had higher BMD levels in the total femur (β = 0.106, P = 0.001, P for trend = 0.001), femur neck (β = 0.106, P = 0.001, P for trend = 0.001), and total spine (β = 0.053, P = 0.009, P for trend = 0.003). When every interested covariate was adjusted, similar significant positive relationships were also found between the BMD in the total femur and femur neck and selenium intake. A similar association was found between total selenium intake and BMD.

**Table 3:** Linear regression coefficients for selenium intakes and BMD.

Intake of selenium (µg/day)	Range of selenium Intake (µg/day)	BMD (gm/cm2)					
		Total femur β (p)		Femoral neck β (p)		Total spine β (p)	
		Model 1^a^	Model 2^b^	Model 1^a^	Model 2^b^	Model 1^a^	Model 2^b^
Dietary selenium (µg/day)							
Q1	≤73.15	Reference	Reference	Reference	Reference	Reference	Reference
Q2	73.16-98.55	0.042(0.020)	0.029(0.101)	0.043(0.024)	0.029(0.114)	0.009(0.640)	0.001(0.991)
Q3	98.56-130.05	0.073(0.001)	0.051(0.004)	0.082(0.001)	0.059(0.002)	0.050(0.010)	0.035(0.072)
Q4	≥130.06	0.106(0.001)	0.069(0.001)	0.106(0.001)	0.064(0.001)	0.053(0.009)	0.030(0.136)
P for trend		0.001	0.001	0.001	0.001	0.003	0.064
Total selenium (µg/day)							
Q1	≤79.35	Reference	Reference	Reference	Reference	Reference	Reference
Q2	79.36-110.05	0.037(0.040)	0.025(0.157)	0.042(0.027)	0.029(0.117)	0.003(0.876)	-0.006(0.739)
Q3	110.06-147.75	0.060(0.001)	0.047(0.008)	0.067(0.001)	0.054(0.004)	0.029(0.142)	0.018(0.353)
Q4	≥147.76	0.087(0.001)	0.060(0.001)	0.086(0.001)	0.058(0.003)	0.047(0.019)	0.030(0.140)
P for trend		0.001	0.001	0.001	0.001	0.008	0.070

a Linear regression adjusted for sex, body mass index, marital status and race.

b Linear regression further adjusted for history of hypertension, history of diabetes, history of osteoporosis in parents , history of previous fractures, education ,age and history of smoking.

### Relation between selenium intake and osteoporosis or BMD in the subgroup analysis by sex

To further demonstrate the relationship between Se intake levels and the risk of OP, we performed a gender-stratified subgroup analysis (**Tables 4**, **5**). The results showed a statistically significant increase in total femur BMD in men and women. However, this relationship was not significantly different between the femoral neck BMD and total spine BMD in men. In women, compared to the lowest quartile (Q1) of Se intake, high Se intake was associated with higher BMD in the femoral neck (all P trend = 0.001). Similar results were obtained when selenium intake was considered to be a continuous variable (per 1-SD increase). Additionally, this positive relationship was significant between the highest and lowest selenium intake quartiles of total spine BMD. According to the logistic regression, the ORs were 0.56 (0.34, 0.91) and 0.62 (0.38, 0.96) in Model 1 and Model 2 for females regarding dietary selenium with OP across quartile 4 relative to quartile 1; however, this association was not significant relative to quartile 1 for men. The highest quartiles of total selenium intake were not significantly related to the risk of osteoporosis in men.

**Table 4:** Association between selenium intake levels and BMD by sex.

Variable	Range of selenium intake (µg/day)	Male		Female	
		Model1^a^	Model 2^b^	Model1^a^	Model2^b^
		β (p)	β (p)	β (p)	β (p)
Total femur BMD( gm/cm2)					
Dietary selenium (µg/day)					
Continuous (Per 1-SD increase)		0.036(0.132)	0.039(0.101)	0.089(0.001)	0.081(0.001)
Q1	≤73.15	Reference		Reference	
Q2	73.16-98.55	0.017(0.590)	0.018(0.578)	0.036(0.131)	0.033(0.165)
Q3	98.56-130.05	0.018(0.600)	0.019(0.571)	0.074(0.001)	0.066(0.004)
Q4	≥130.06	0.072(0.040)	0.078(0.025)	0.083(0.001)	0.078(0.001)
P for trend		0.021	0.011	0.001	0.001
Total selenium (µg/day)					
Continuous (Per 1-SD increase)		0.037(0.117)	0.039(0.104)	0.074(0.001)	0.063(0.003)
Q1	≤79.35	Reference		Reference	
Q2	79.36-110.05	0.026(0.410)	0.031(0.328)	0.030(0.210)	0.024(0.307)
Q3	110.06-147.75	0.048(0.143)	0.051(0.116)	0.057(0.016)	0.048(0.045)
Q4	≥147.76	0.068(0.047)	0.074(0.032)	0.084(0.001)	0.074(0.001)
P for trend		0.044	0.034	0.001	0.001
Femoral neck BMD( gm/cm2)					
Dietary selenium (µg/day)					
Continuous (Per 1-SD increase)		0.023(0.325)	0.024(0.300)	0.082(0.001)	0.078(0.001)
Q1	≤73.15	Reference		Reference	
Q2	73.16-98.55	0.015(0.647)	0.016(0.610)	0.035(0.142)	0.035(0.140)
Q3	98.56-130.05	0.030(0.364)	0.031(0.343)	0.068(0.003)	0.063(0.001)
Q4	≥130.06	0.056(0.103)	0.061(0.081)	0.082(0.001)	0.080(0.007)
P for trend		0.072	0.055	0.001	0.001
Total selenium (µg/day)					
Continuous (Per 1-SD increase)		0.024(0.311)	0.024(0.312)	0.069(0.001)	0.063(0.003)
Q1	≤79.35	Reference		Reference	
Q2	79.36-110.05	0.018(0.557)	0.021(0.503)	0.037(0.127)	0.035(0.143)
Q3	110.06-147.75	0.051(0.115)	0.053(0.100)	0.058(0.015)	0.052(0.031)
Q4	≥147.76	0.045(0.184)	0.047(0.165)	0.084(0.001)	0.079(0.001)
P for trend		0.192	0.189	0.001	0.001
Total spine BMD( gm/cm2)					
Dietary selenium (µg/day)					
Continuous (Per 1-SD increase)		0.021(0.398)	0.020(0.423)	0.055(0.013)	0.046(0.035)
Q1	≤73.15	Reference		Reference	
Q2	73.16-98.55	-0.006(0.860)	-0.008(0.814)	0.001(0.982)	-0.006(0.812)
Q3	98.56-130.05	0.002(0.026)	-0.002(0.964)	0.042(0.081)	0.034(0.160)
Q4	≥130.06	0.033(0.360)	0.031(0.389)	0.066(0.008)	0.059(0.017)
P for trend		0.203	0.215	0.014	0.034
Total selenium (µg/day)					
Continuous (Per 1-SD increase)		0.030(0.217)	0.028(0.260)	0.041(0.066)	0.030(0.169)
Q1	≤79.35	Reference		Reference	
Q2	79.36-110.05	-0.004(0.892)	-0.007(0.832)	-0.002(0.937)	-0.008(0.741)
Q3	110.06-147.75	0.015(0.665)	0.011(0.745)	0.030(0.229)	0.022(0.391)
Q4	≥147.76	0.043(0.228)	0.039(0.273)	0.052(0.032)	0.043(0.075)
P for trend		0.108	0.130	0.018	0.051

a Model 1 is shown as the odds ratio (95% confidence interval); adjusted for sex, body mass index, marital status and race.

b Model 2 is shown as an odds ratio (95% confidence interval); further adjusted for history of hypertension, history of diabetes, history of osteoporosis in parents and history of previous fractures.

c Model 3 is shown as odds ratio (95% confidence interval); further adjusted for education ,age and history of smoking.

**Table 5:** Association between selenium intake levels and osteoporosis by sex.

Variable	Range of selenium intake (µg /day)	Male				Female			
		Model 1^a^		Model 2^b^		Model 1^a^		Model 2^b^	
		OR (95% CI)	P-value	OR (95% CI)	P-value	OR (95% CI)	P-value	OR (95% CI)	P-value
Dietary selenium (µg/day)									
Continuous (Per 1-SD increase)		1.000(0.994,1.006)	0.980	1.002(0.996,1.007)	0.585	0.995(0.991,0.999)	0.009	0.996(0.992,1.000)	0.030
Q1	≤73.15	Reference		Reference		Reference		Reference	
Q2	73.16-98.55	0.899(0.370,2.185)	0.814	1.035(0.143,2.591)	0.942	0.849(0.594,1.212)	0.367	0.867(0.603,1.247)	0.442
Q3	98.56-130.05	1.033(0.448,2.381)	0.939	1.185(0.497,2.827)	0.702	0.708(0.474.1.058)	0.092	0.729(0.485,1.097)	0.129
Q4	≥130.06	0.567(0.224,1.435)	0.231	0.741(0.281,1.955)	0.544	0.560(0.344,0.911)	0.020	0.617(0.376,0.961)	0.045
P for trend		0.229		0.534		0.002		0.031	
Total selenium (µg/day)									
Continuous (Per 1-SD increase)		0.997(0.992,1.003)	0.330	0.999(0.994,1.004)	0.697	0.997(0.994,1.000)	0.036	0.997(0.994,1.001)	0.110
Q1	≤79.35	Reference		Reference		Reference		Reference	
Q2	79.36-110.05	1.238(0.491,3.122)	0.651	1.435(0.546,3.771)	0.463	0.971(0.692,1.362)	0.865	0.962(0.666,1.390)	0.838
Q3	110.06-147.75	1.608(0.696,3.715)	0.267	1.976(0.818,4.770)	0.130	0.676(0.462,0.988)	0.043	0.716(0.475,1.080)	0.111
Q4	≥147.76	0.541(0.203,1.439)	0.218	0.720(0.256,2.023)	0.533	0.803(0.521,1.236)	0.318	0.709(0.442,1.139)	0.155
P for trend		0.139		0.412		0.027		0.074	

a Model 1 is shown as the odds ratio (95% confidence interval); adjusted for body mass index, age, race, marital status, history of osteoporosis in parents and history of previous fractures.

b Model 2 is shown as an odds ratio (95% confidence interval); further adjusted for history of hypertension, history of diabetes, education and history of smoking.

## Discussion

Few studies have investigated whether diet and total selenium intake are associated with bone health. Based on US adult samples from NHANES (2009–2010, 2013–2014, and 2017–2020) in this study, we found that selenium intake was independently related to a lower risk of OP, while a higher selenium intake was related to a higher total femur/femoral neck BMD. Additionally, higher Se intake among females was related to a higher total femur/femoral neck BMD in women, but not in men.

This is the first study to determine the correlation between selenium intake and factors such as BMD and the risk of osteoporosis in adults in the US based on large data. Although some studies have investigated the effects of dietary selenium intake on bone health, and those that have are limited by small sample sizes (19, 20, 24). In our study, we analyzed the relationship between selenium intake and bone health from 3 cycles of NHANES (2009-2010, 2013-2014, and 2017-2020) in middle-aged and older adults aged 50 years or older, overcoming the small amount of data described in previous studies. In addition, studies have investigated the relationship between selenium intake and BMD of adults in the US and other countries (19, 20, 24–26), but the results of their studies were different. For example, Wolf et al. suggested in their cross-sectional study that Se intake from the diet did not have any association with BMD in the US adults (24); Xue et al. showed that an increase in the dietary Se level can predict higher BMD levels in the femur, femur neck, intertrochanter, trochanter, and that dietary selenium intake had an inverted U-shaped relationship with bone mineral density in the US adults (20); Walsh et al. conducted a randomized double-blinded controlled study and found that Se intake at 200 µg/day did not significantly alter the musculoskeletal health of postmenopausal women in the UK (25); A cross-sectional study conducted with 6,267 participants found that a higher dietary intake of Se increased the BMD of individuals in a middle-aged and elderly Chinese population (19); Recently, Xie et al. showed that selenium was positively associated with BMD and inversely associated with OP by a meta-analysis of data from different countries (26); Thus, it is necessary to further clarify the relationship between dietary selenium and bone mineral density in the US adults. Furthermore, previous study (20) have only analyzed the relationship between dietary selenium intake and bone mineral density, and did not consider selenium intake from dietary supplements. In our study, in addition to analyzing the relationship between dietary selenium intake and bone mineral density, we also analyzed the relationship between total selenium intake (dietary selenium intake and supplement intake from dietary supplements) and bone mineral density, so our study is more comprehensive and detailed than previous studies. We results showed that selenium intake was positively associated with total femur/femoral neck BMD, which partially matched the findings of previous study, supporting the finding that selenium intake is positively related to BMD. Meanwhile, very few articles have investigated the relationship between the status of selenium and osteoporosis in the US population. Although Zhang et al. suggested that Se intake was negatively associated with the risk of osteoporotic hip fracture, those researchers focused on smokers (27). Xue et al. showed an inverted U-shaped relationship between dietary selenium intake and bone mineral density, but they did not assess the relationship between dietary selenium intake and osteoporosis risk due to limitations of the original data (26). This is the first analysis between the dietary and total intake of selenium and osteoporosis among US adults. Our results indicate that dietary and total selenium intake was negative with the risk of osteoporosis in US adults.

Selenium is an essential trace mineral element in the human body. It can regulate cellular processes, such as the Se-driven antioxidant enzyme component, which can scavenge ROS in cells (28–30). The mechanism by which selenium plays a role in the development of osteoporosis is not clear, but we hypothesized several mechanisms that link selenium and osteoporosis. First, ROS has a critical effect on the development of OP (16). Apoptosis of osteoblasts and osteocytes induced by ROS may promote the production of osteoclasts and inhibit osteogenesis and mineralization. However, excessive osteocyte apoptosis can result in oxidative stress, which can disrupt the generation of osteoclasts, increase bone loss, and result in remodeling (31, 32). Selenoproteins are transporters of Se in bones, and they are found in osteoclasts and osteoblasts. They have antioxidant activities and can scavenge the generated ROS (15, 16, 33). Second, selenium can also influence anti-inflammatory and immune processes. Since cytokines such as interleukin-6 (IL-6) are important for the pathogenic mechanism of OP. Selenium exerts an anti-inflammatory effect, partially regulated via the inhibition of cytokine activities; thus, Se regulates bone turnover to protect against OP (34, 35). Third, Se-mediated iodothyronine deiodinases help regulate thyroid hormone turnover, while Se-mediated glutathione peroxidases help in protecting the thyroid gland. Thus, Se might also affect bone health via the relationship between Se-mediated glutathione peroxidase activity and thyroid protection. A deficiency of Se might increase the blood thyroid hormone level, thus accelerating bone loss and osteoporosis (36, 37). Therefore, determining the relationship between selenium intake and OP is extremely important.

This study had some strengths. First, this study was conducted based on a nationwide survey, where BMD was determined using established approaches by expert scientists. Second, this was the first study to analyze the relationship between Se intake and parameters such as BMD and OP among adults in the US. Our results showed a potential role of appropriate dietary Se intake in preventing the development of OP. Additionally, after confounding factors were adjusted, a gender-stratified subgroup analysis was conducted to provide a reference to determine the relationship between selenium intake and changes in BMD and risk of osteoporosis. However, our study had certain limitations. First, this was a cross-sectional study, and residual confounding due to additional unmeasured factors was not eliminated, although certain covariates were adjusted. Second, we acquired dietary intake information from the NHANES based on 24-h dietary recall interviews, which might be associated with recall bias. Hence, our results should be confirmed by conducting larger, prospective studies.

To summarize, the level of dietary and total Se intake was positively associated with BMD and negatively associated with the risk of OP among adults in the US. However, larger prospective studies are needed to confirm our findings.

## Data availability statement

The original contributions presented in the study are included in the article/supplementary material. Further inquiries can be directed to the corresponding authors.

## Ethics statement

The studies involving humans were approved by the Institutional Review Board of the National Center for Health Statistics. The studies were conducted in accordance with the local legislation and institutional requirements. The participants provided their written informed consent to participate in this study.

## Author contributions

SP analyzed the data, and wrote the manuscript. GZ and DW designed the study and revised the manuscript. SP is the first author. All authors contributed to the article and approved the submitted version.
